# Supplementary material for: Resolving the full spectrum of human genome variation using Linked-Reads
Source: Genome Res. 2019 Apr;29(4):635–45. doi: 10.1101/gr.234443.118 (PMC6442396; doi:10.1101/gr.234443.118)
Supplement: Supplemental Material [file supp_gr.234443.118_Supplemental_longranger-2.2.zip › longranger-2.2.tar.bz2!/lr2.2-public/lib/stan/cmdstan/stan/doc/api/html/structstan_1_1gm_1_1local__var__decl__visgen.html]

Stan: stan::gm::local\_var\_decl\_visgen Struct Reference


|  |  |
| --- | --- |
|  | Stan  2.5.0  probability, sampling & optimization |


- Main Page
- Related Pages
- Namespaces
- Classes
- Files

- Class List
- Class Index
- Class Hierarchy
- Class Members

All Classes Namespaces Files Functions Variables Typedefs Enumerations Enumerator Friends Macros Pages

- stan
- gm
- local\_var\_decl\_visgen

Public Member Functions |
Public Attributes |
List of all members

stan::gm::local\_var\_decl\_visgen Struct Reference

`#include <generator.hpp>`

Inheritance diagram for stan::gm::local\_var\_decl\_visgen:

|  |  |
| --- | --- |
| Public Member Functions | |
|  | local\_var\_decl\_visgen (int indents, bool is\_var, bool is\_fun\_return, std::ostream &o) |
|  | |
| void | operator() (nil const &) const |
|  | |
| void | operator() (int\_var\_decl const &x) const |
|  | |
| void | operator() (double\_var\_decl const &x) const |
|  | |
| void | operator() (vector\_var\_decl const &x) const |
|  | |
| void | operator() (row\_vector\_var\_decl const &x) const |
|  | |
| void | operator() (matrix\_var\_decl const &x) const |
|  | |
| void | operator() (unit\_vector\_var\_decl const &x) const |
|  | |
| void | operator() (simplex\_var\_decl const &x) const |
|  | |
| void | operator() (ordered\_var\_decl const &x) const |
|  | |
| void | operator() (positive\_ordered\_var\_decl const &x) const |
|  | |
| void | operator() (cholesky\_factor\_var\_decl const &x) const |
|  | |
| void | operator() (const cholesky\_corr\_var\_decl &x) const |
|  | |
| void | operator() (cov\_matrix\_var\_decl const &x) const |
|  | |
| void | operator() (corr\_matrix\_var\_decl const &x) const |
|  | |
| void | generate\_type (const std::string &type, size\_t num\_dims) const |
|  | |
| void | generate\_void\_statement (const std::string &name) const |
|  | |
| void | generate\_init\_args (const std::string &type, const std::vector< expression > &ctor\_args, const std::vector< expression > &dims, size\_t dim) const |
|  | |
| void | declare\_array (const std::string &type, const std::vector< expression > &ctor\_args, const std::string &name, const std::vector< expression > &dims) const |
|  | |
| Public Member Functions inherited from stan::gm::visgen | |
|  | visgen (std::ostream &o) |
|  | |

|  |  |
| --- | --- |
| Public Attributes | |
| int | indents\_ |
|  | |
| bool | is\_var\_ |
|  | |
| bool | is\_fun\_return\_ |
|  | |
| Public Attributes inherited from stan::gm::visgen | |
| std::ostream & | o\_ |
|  | |

|  |  |
| --- | --- |
| Additional Inherited Members | |
| Public Types inherited from stan::gm::visgen | |
| typedef void | result\_type |
|  | |

## Detailed Description

Definition at line 996 of file generator.hpp.

## Constructor & Destructor Documentation

|  |  |  |  |
| --- | --- | --- | --- |
| stan::gm::local\_var\_decl\_visgen::local\_var\_decl\_visgen | ( | int | *indents*, |
|  |  | bool | *is\_var*, |
|  |  | bool | *is\_fun\_return*, |
|  |  | std::ostream & | *o* |
|  | ) |  |  |

inline

Definition at line 1000 of file generator.hpp.

## Member Function Documentation

|  |  |  |  |
| --- | --- | --- | --- |
| void stan::gm::local\_var\_decl\_visgen::declare\_array | ( | const std::string & | *type*, |
|  |  | const std::vector< expression > & | *ctor\_args*, |
|  |  | const std::string & | *name*, |
|  |  | const std::vector< expression > & | *dims* |
|  | ) |  | const |

inline

Definition at line 1189 of file generator.hpp.

|  |  |  |  |
| --- | --- | --- | --- |
| void stan::gm::local\_var\_decl\_visgen::generate\_init\_args | ( | const std::string & | *type*, |
|  |  | const std::vector< expression > & | *ctor\_args*, |
|  |  | const std::vector< expression > & | *dims*, |
|  |  | size\_t | *dim* |
|  | ) |  | const |

inline

Definition at line 1142 of file generator.hpp.

|  |  |  |  |
| --- | --- | --- | --- |
| void stan::gm::local\_var\_decl\_visgen::generate\_type | ( | const std::string & | *type*, |
|  |  | size\_t | *num\_dims* |
|  | ) |  | const |

inline

Definition at line 1124 of file generator.hpp.

|  |  |  |  |  |  |
| --- | --- | --- | --- | --- | --- |
| void stan::gm::local\_var\_decl\_visgen::generate\_void\_statement | ( | const std::string & | *name* | ) | const |

inline

Definition at line 1135 of file generator.hpp.

|  |  |  |  |  |  |
| --- | --- | --- | --- | --- | --- |
| void stan::gm::local\_var\_decl\_visgen::operator() | ( | nil const & |  | ) | const |

inline

Definition at line 1009 of file generator.hpp.

|  |  |  |  |  |  |
| --- | --- | --- | --- | --- | --- |
| void stan::gm::local\_var\_decl\_visgen::operator() | ( | int\_var\_decl const & | *x* | ) | const |

inline

Definition at line 1010 of file generator.hpp.

|  |  |  |  |  |  |
| --- | --- | --- | --- | --- | --- |
| void stan::gm::local\_var\_decl\_visgen::operator() | ( | double\_var\_decl const & | *x* | ) | const |

inline

Definition at line 1014 of file generator.hpp.

|  |  |  |  |  |  |
| --- | --- | --- | --- | --- | --- |
| void stan::gm::local\_var\_decl\_visgen::operator() | ( | vector\_var\_decl const & | *x* | ) | const |

inline

Definition at line 1021 of file generator.hpp.

|  |  |  |  |  |  |
| --- | --- | --- | --- | --- | --- |
| void stan::gm::local\_var\_decl\_visgen::operator() | ( | row\_vector\_var\_decl const & | *x* | ) | const |

inline

Definition at line 1029 of file generator.hpp.

|  |  |  |  |  |  |
| --- | --- | --- | --- | --- | --- |
| void stan::gm::local\_var\_decl\_visgen::operator() | ( | matrix\_var\_decl const & | *x* | ) | const |

inline

Definition at line 1039 of file generator.hpp.

|  |  |  |  |  |  |
| --- | --- | --- | --- | --- | --- |
| void stan::gm::local\_var\_decl\_visgen::operator() | ( | unit\_vector\_var\_decl const & | *x* | ) | const |

inline

Definition at line 1050 of file generator.hpp.

|  |  |  |  |  |  |
| --- | --- | --- | --- | --- | --- |
| void stan::gm::local\_var\_decl\_visgen::operator() | ( | simplex\_var\_decl const & | *x* | ) | const |

inline

Definition at line 1058 of file generator.hpp.

|  |  |  |  |  |  |
| --- | --- | --- | --- | --- | --- |
| void stan::gm::local\_var\_decl\_visgen::operator() | ( | ordered\_var\_decl const & | *x* | ) | const |

inline

Definition at line 1066 of file generator.hpp.

|  |  |  |  |  |  |
| --- | --- | --- | --- | --- | --- |
| void stan::gm::local\_var\_decl\_visgen::operator() | ( | positive\_ordered\_var\_decl const & | *x* | ) | const |

inline

Definition at line 1074 of file generator.hpp.

|  |  |  |  |  |  |
| --- | --- | --- | --- | --- | --- |
| void stan::gm::local\_var\_decl\_visgen::operator() | ( | cholesky\_factor\_var\_decl const & | *x* | ) | const |

inline

Definition at line 1082 of file generator.hpp.

|  |  |  |  |  |  |
| --- | --- | --- | --- | --- | --- |
| void stan::gm::local\_var\_decl\_visgen::operator() | ( | const cholesky\_corr\_var\_decl & | *x* | ) | const |

inline

Definition at line 1093 of file generator.hpp.

|  |  |  |  |  |  |
| --- | --- | --- | --- | --- | --- |
| void stan::gm::local\_var\_decl\_visgen::operator() | ( | cov\_matrix\_var\_decl const & | *x* | ) | const |

inline

Definition at line 1102 of file generator.hpp.

|  |  |  |  |  |  |
| --- | --- | --- | --- | --- | --- |
| void stan::gm::local\_var\_decl\_visgen::operator() | ( | corr\_matrix\_var\_decl const & | *x* | ) | const |

inline

Definition at line 1113 of file generator.hpp.

## Member Data Documentation

int stan::gm::local\_var\_decl\_visgen::indents\_

Definition at line 997 of file generator.hpp.

bool stan::gm::local\_var\_decl\_visgen::is\_fun\_return\_

Definition at line 999 of file generator.hpp.

bool stan::gm::local\_var\_decl\_visgen::is\_var\_

Definition at line 998 of file generator.hpp.

---

The documentation for this struct was generated from the following file:

- src/stan/gm/generator.hpp


---

[ Stan Home Page ]

*© 2011–2014,
Stan Development Team.*
